# Sprint Interval Training Improves Brain-Derived Neurotropic Factor-Induced Benefits in Brain Health—A Possible Molecular Signaling Intervention

**DOI:** 10.3390/biology13080562

**Published:** 2024-07-26

**Authors:** Xueqiang Zhu, Wenjia Chen, Anand Thirupathi

**Affiliations:** 1 School of Competitive Sports, Shandong Sport University, Rizhao 276826, China; 2 School of Physical Education, China University of Mining and Technology, Xuzhou 221116, China; richabloomer@hotmail.com; 3 Faculty of Sports Science, Ningbo University, Ningbo 315211, China; anand@nbu.edu.cn

**Keywords:** sprint interval training, intensity, BDNF, TrkB, molecular signaling

## Abstract

**Simple Summary:**

Advances in exercise science have revealed how physical exercise performance can help to shape our physiology. However, individual genetic traits, physical characteristics, and different exercise protocols can affect each molecular target to achieve exercise-induced benefits, and this challenge causes exercise to be prescribed as standard therapy for several pathological conditions. Therefore, each exercise protocol must be thoroughly investigated. Sprint interval training (SIT) influences the brain-derived neurotrophic factor (BDNF) response for maintaining brain health through several possible forms of molecular signaling, which leads to crosstalk that can improve BDNF response. In addition, regulating BDNF levels can provide a protective effect on bipolar disorders since BDNF elevation is associated with bipolar disorders. For example, SNP rs6265 is a single nucleotide substitution that disrupts BDNF secretion and transports and affects its functions to cause neurocognitive impairments. Moderate intensity possesses a protective effect in reducing depression by impacting SNP rs6265 for BDNF secretion, while high-intensity exercise improves locomotor learning by influencing SNP rs6265. This is crucial in terms of reducing depression, schizophrenia, and bipolar disorders and improving mood and cognitive functions. Although no studies have established the link between these genetic variants by SIT protocols for BDNF regulation, HIT protocols are considered to be the same as SIT, which can support SIT, which may affect this genetic variant for BDNF regulation. Moreover, BDNF induces a fatigue mechanism by increasing beta-hydroxybutyrate. This could affect the individual’s SIT performance and mood.

**Abstract:**

Physical exercise can significantly impact our bodies, affecting our functional capacity, structure establishment, and molecular makeup. The magnitude of these changes depends on the specific exercise protocols used. For instance, low-to-moderate-intensity exercise can activate important molecular targets in the short term, such as BDNF-mediated signaling, while high-intensity exercise can maintain these signaling molecules in the active state for a longer term. This makes it challenging to recommend specific exercises for obtaining BDNF-induced benefits. Additionally, exercise-induced molecular signaling targets can have positive and negative effects, with some exercises blunting these targets and others activating them. For example, increasing BDNF concentration through exercise can be beneficial for brain health, but it may also have a negative impact on conditions such as bipolar disorder. Therefore, a deeper understanding of a specific exercise-mediated mechanistic approach is required. This review will delve into how the sprint exercise-mediated activation of BDNF could help maintain brain health and explore potential molecular interventions.

## 1. Introduction

The health benefits of physical exercise are well known, but the specific dose–response determines how local organs achieve systemic effects [1]. This requires various molecular landscape changes either within a specific cell or changes in a specific cell that induce a pleiotropic effect on the neighboring cell [1,2]. Various molecules, such as enzymes, neurotrophins, and receptors, are involved during these physiological actions [3,4]. One of the neurotrophic factors, brain-derived neurotrophic factor (BDNF), can support these physiological actions, particularly in shaping long-term brain physiology [5]. Since BDNF supports the differentiation, proliferation, and survival of neurons in the brain in many severe conditions, including cerebral ischemia and neurotoxicity, it is important to understand how the BDNF protein is stimulated or maintained for normal physiological conditions in the brain. In addition, BDNF may have a significant impact on stimulating angiogenesis, enhancing endothelial cell survival, and preserving vascular integrity [6]. BDNF is generally expressed in the brain, mainly in the hippocampus, cortex, and basal forebrain areas, as well as skeletal muscle, retina, kidneys, and prostate [7]. 

The effects of BDNF are mediated by various intracellular signaling pathways. For instance, BDNF activates the ERK/CREB and PI3/Akt pathways, as well as the phospholipase Cγ pathway, to support neuronal survival. ERK activation by BDNF can enhance the expression of pre- and post-synaptic proteins [8]. Furthermore, BDNF regulates the release of neurotransmitters, such as glutamate, in the cortical neurons through the activation of the PLCγ signaling pathway. Additionally, the p75 receptor, which has a low affinity for BDNF, mediates hippocampal homeostatic plasticity through BDNF, ERK, and the RhoA-ROCK2-LIMK1-cofilin signaling pathway [9]. Nevertheless, BDNF regulates the local mRNA translation for synaptic plasticity, mainly via Ca^2+^-calmodulin-dependent protein kinase II signaling, Homer,2 in the synaptodendritic compartment through the mTOR-PI3 signaling pathway [10]. Since muscle cells are metabolically active, they can release several molecules, including BDNF, which is crucial for exercise-induced brain benefits. For example, the activation of the AMPK signaling pathway increases cathepsin B secretion in the muscle, and it can cross the blood–brain barrier (BBB) to induce BDNF [11]. This scenario is important for neuronal migration and neurogenesis. The elevation of PGC-1α induces FNDC5 expression, which increases irisin secretion in the blood and passes through the BBB for BDNF secretion in the hippocampus [11]. Next, muscle BDNF is increased by activating JNK and NF-kB signaling pathways [12]. However, activating this molecule requires an effective dose response to exercise [13]. 

Interval training has been used for many years to improve health parameters and performance. It involves performing high-intensity exercise for shorter periods followed by low or rest periods. Although studies have reported the effect of HIT on BDNF and brain health, no studies have reported the effect of SIT on BDNF regulation. Even though HIT protocols are often considered similar to SIT, SIT protocols have higher intensity and shorter rest periods [14], which is crucial for faster mitochondrial adaptation and mitochondrial biogenesis via activating PGC-1α. Therefore, it is important to thoroughly investigate each protocol to gain better knowledge of how BDNF is regulated by SIT. This understanding is crucial for improving brain function and tailoring these protocols for specific populations. Indeed, several studies reported that HIT exercises, such as running or cycling, could be the most obvious forms of exercise in activating BDNF-induced benefits [14,15]. Therefore, athletes and people who want to obtain exercise-induced benefits within shorter periods may prefer HIT protocols [15]. To achieve this, sprint techniques have emerged, such as repeated sprint training (lasting from 3 to 7 s) or sprint interval training (SIT) (30 s to 2–4 min) [2]. Nevertheless, few studies have established the link between SIT and BDNF in brain health (Table 1). 

In the 1920s, Hill conducted groundbreaking experiments that included intermittent exercises, laying the foundation for our understanding of HIT. Since then, numerous studies have reported on acute physiological responses to HIT [22,23], forming the initial scientific framework for long- and short-duration intervals and revealing almost all the physiological responses induced by HIT. However, how these physiological responses are orchestrated by molecular signaling is unknown in many cases, especially under pathological conditions, rendering the recommendation of exercise like HIT in neurodegenerative conditions, including Parkinson’s or Alzheimer’s diseases, uncertain [24]. However, a recent study showed that vigorous cycling exercise for six min increased BDNF levels four-to-five-fold (396 pg L^−1^ to 1170 pg L^−1^) when compared to light intensity for 90 min or combined fasting and exercise, possibly switching to the use of lactate as a primary fuel rather than glucose in the cerebral brain, which elevates BDNF levels [25]. Furthermore, exercise intensity also leads to an increase in platelet numbers, which store a significant amount of BDNF. This can potentially enhance learning and memory and offer protection against an age-related cognitive decline caused by diseases like Alzheimer’s and Parkinson’s [25,26]. Indeed, low-to-moderate-intensity training failed to activate some important molecular targets [16], while HIT activated signaling through adaptation [27,28]. As mentioned, BDNF is one of the molecular targets from exercise that produce various benefits in the brain, such as neuronal plasticity, neuronal survival (proliferation and differentiation) [27], increased dopamine [29], improved memory [30], and influencing genomic alteration for local protein synthesis in a specific neuron region [29,30]. By activating all these responses, BDNF requires a stimulator to activate BDNF directly or its upstream targets. However, most of the time, exercise protocols with low intensity ambiguously fail to improve the BDNF response and are reluctant to prescribe exercise as a non-pharmacological tool for enhancing BNDF-induced benefits, especially in alleviating neurodegenerative diseases like Parkinson’s or Alzheimer’s [31].

## 2. SIT-Mediated Molecular Signaling on BDNF’s Response in the Muscle

The moderate intensity of different exercise types temporarily improves motor control and cognitive function by facilitating the interaction of peripheral nerves in the skeletal muscle at the neuromuscular junction (NMJ) [32]. Consequently, it improves synapse preservation by altering synaptic element morphology and increases fast-to-slow fiber transitions. This is primarily accomplished through BDNF/TrkB signaling [33]. However, all these effects are reversed with detraining [34,35], suggesting the requirement of vigorous-intensity performances like SIT, which can maintain BDNF/ TrkB-mediated signaling in the skeletal muscle to sustain these effects for the longer term. Furthermore, exercise like SIT affects the interaction of BDNF isoforms for NMJ function in the skeletal muscle [36,37]. Studies have shown that the full-length isoform of TrkB activates the presynaptic protein kinase C (PKC) by phosphoinositide-dependent kinase 1 (PDK1), which alters Acetylcholine (Ach) release by mammalian uncoordinated-18 (Munc18-1) and synaptosomal nerve-associated protein 25 phosphorylation (SNAP-25) for promoting neuromuscular transmission [35,38,39].

## 3. SIT-Mediated Molecular Signaling on BDNF’s Response in the Brain

**Figure 1:**
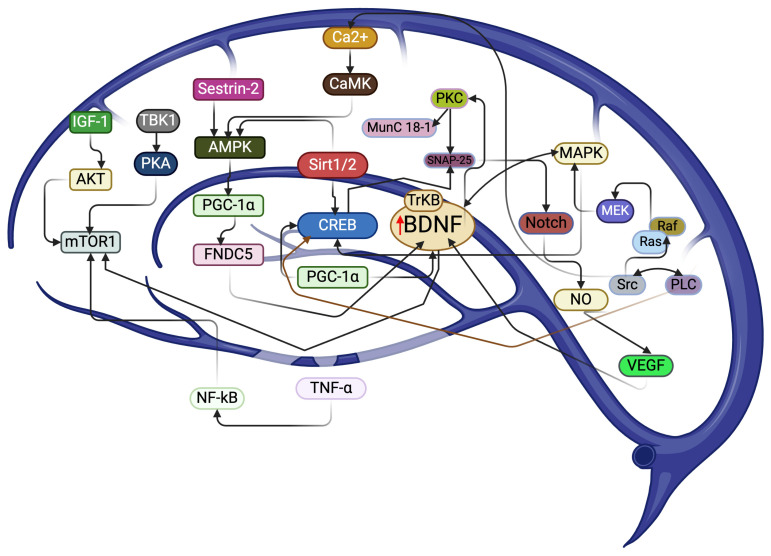
A possible molecular target induced by SIT for BDNF response in maintaining brain health. BDNF-induced response can phosphorylate PKC to increase MunC 18-1 and SNAP-25, while the CREB transcription factor can activate SNAP-25 for BDNF response. The modulation of calcium signaling by SIT can activate AMPK, which could activate PGC-1α-induced FNDC5 for BDNF response. AMPK-mediated Notch signaling by SIT could activate NO and VEGF for BDNF response. PLCγ-mediated signaling activated by SIT can activate Src, Ras/Raf, and MEK for MAPK activation, consequently improving the BDNF level. SIT-activated IGF-1 can cause mTOR activation through AKT signaling for the BDNF response. TNF-α and NF-kB can also activate mTOR signaling for the BDNF response by SIT.

The cAMP response element-binding protein (CREB) is a transcription factor that can transcribe various genes, including BDNF [39]. For example, CREB phosphorylates SNAP-25 to activate TrkB/BDNF signaling in the brain [39,40] in an exercise-intensity manner. For instance, SIT with an intensity of 80–100% VO2 max increases the BDNF level and improves depression and anxiety [18] by crosstalk with PKA/Akt/CREB and MAPK/CREB pathways, especially when mediated by the estrogen receptors in the female hippocampus [41]. However, whether male hormones like testosterone influence the levels of BDNF during SIT needs to be explored. A study has shown that functional fitness training can increase the serum BDNF level independent of male hormone testosterone in a dose-dependent manner [42]. Moreover, PDK1 activates RSK1/2 to facilitate BDNF response for neuronal survival [43] (Table 2). Specifically, PDK1 phosphorylates PKC, which subsequently phosphorylates Mun18-1 and SNAP-25 for Acetylcholine release and synaptic secretion [44], mediated by BDNF/TrkB in an exercise type-dependent manner (Figure 1) [32]. A recent study revealed that running with moderate intensity activates this pathway, while swimming exercise has the opposite effect on the muscles [32]. Nonetheless, further exploration is warranted with SIT protocols. In addition, oxidative stress and inflammation may also contribute to an increase in the BDNF during SIT. This is evidenced by an increase in hydrogen peroxide and tumor necrosis factor-alpha (TNF-α) during SIT in the brain [45]. This increase is mediated by the translocation of p65:p50 of nuclear factor kappa B (NF-κB) from the cytoplasm to the nucleus to activate the CREB transcription factor [45]. Consequently, it activates the mitochondrial biogenesis protein peroxisome proliferator-activated receptor gamma coactivator 1-alpha (PGC-1α) for BDNF-mediated benefits in the brain [46,47].

In addition, the SIT-induced elevation of lactate could compensate for the energy requirement in the brain regarding neuronal activity [48]. For example, SIT-induced redox flux, due to the elevation of lactate in the neuronal cell, could activate various redox proteins, such as sirtuin (SIRT1) mediated by the NAD+/NADH ratio flux [49], following the activation of PGC-1α/fibronectin type III domain-containing protein 5 (FNDC5), which could mediate BDNF-induced benefits in the brain [50,51]. Moreover, SIT improves cerebral angiogenesis by increasing lactate receptor hydroxycarboxylic acid receptor 1 (HCAR1), which is a key regulator of the vascular endothelial growth factor (VEGR) [52], in addition to improving insulin growth factor-1 (IGF-1) mRNA expression via somatotropic axis stimulation [53]. This can improve cognitive function by increasing lactate metabolism during SIT protocols.

Furthermore, SIT-induced short-term fatigue can accumulate metabolites such as organic phosphate, ADP, and H^+^ [54]. This accumulation can affect the formation of cross-bridges and force production in the muscles, leading to a reduction in calcium flux [54]. As a result, the muscle BDNF concentration decreases, affecting initial BDNF circulation and secretion by Schwann cells [55]. Several molecular signaling pathways mediate the BDNF level, including Ras/MAPK, PI3K/3, PDK1/AKT, and PLC-γ, which may be involved in this physiological adaptation, potentially inducing a survival mechanism in neural cells and promoting local genomic stability in the axon region [56,57]. It is currently unknown whether the muscle BDNF induced by SIT can cross the BBB to provide the benefits associated with BDNF in the brain. Exploring this could shed light on the potential impact of short-term exercises like SIT on improving brain health and preventing neurodegenerative diseases.

## 4. Antagonistic Effects of BDNF on SIT-Induced Signaling

**Figure 2:**
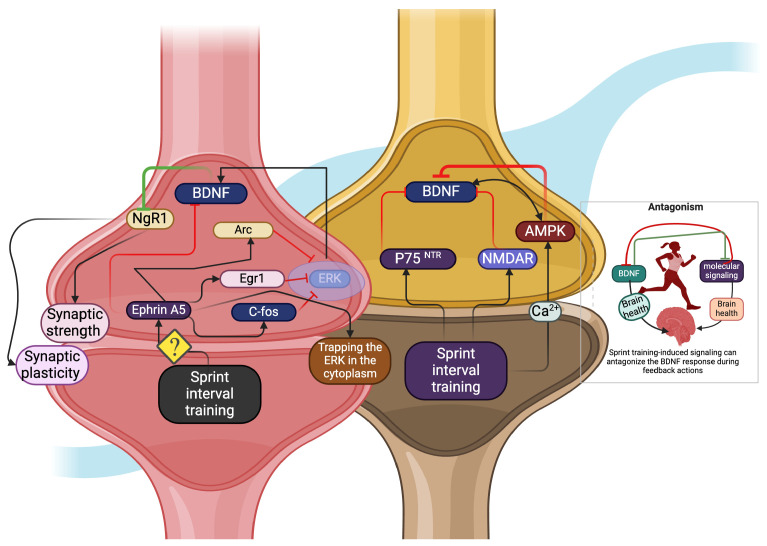
Antagonistic effect of BDNF for SIT-induced signaling. Ephrin A5 (black arrow) antagonizes the BDNF response through C-Fos, Egr1, and Arc (red arrow), which traps ERK in the cytoplasm and consequently decreases the ERK-induced BDNF level. BDNF hinders the NgR1 (Green inhibitory symbol), which prevents NgR1-induced synaptic strength and plasticity, and SIT-mediated signaling like AMPK, NMDAR, and p_75_^NTR^ (black arrow), which can inhibit the BDNF response in the brain (red inhibitory symbol).

High-energy deprivation due to SIT may sensitize the AMP-activated protein kinase (AMPK) molecule, countering the BDNF response in cortical neurons [58]. For example, SIT-induced signaling like sestrin 2 could stabilize the liver kinase B-1 (LKB-1) and AMPK complex by increasing Thr172 phosphorylation [59,60,61], leading to the upregulation of the PGC-1α-induced BDNF response. On the other hand, sestrin-induced TORC2/AKT activation inhibits PGC-1α (Table 2) [62], which may have negative effects on BDNF response. Other signaling molecules, such as ephrin-A5, can also antagonize BDNF-induced neuronal motility by trapping extracellular signal-regulated kinases (ERK) in the cytoplasm through several signaling pathways, such as c-Fos, Egr1, and Arc (Figure 2) [63]. For example, heavy resistance training at 80–85% upregulates the ERK in the muscle [64]. This may bypass the signaling for BDNF response. Furthermore, Nogo receptor-1 (NgR1) signaling regulates synaptic strength and plasticity. A study has shown that NgR1 declines initially with the heavy intensity of running exercise in the brain [65]. However, the extended duration of this exercise in the later stages increases the NgR1 to improve neuronal plasticity [65]. In this case, BDNF can counteract the function of NgR1 [66,67,68]. Additionally, pro-BDNF can activate the postsynaptic p75^NTR^ to decrease synaptic strength. Studies have shown that both aerobic and resistance training protocols can increase the activity of p75^NTR^ [68] and NMDA receptors [69]. For example, intensity with 85–95% treadmill running increases the activity of p75^NTR^ and improves neuroplasticity [70]. This can antagonize the effects of BDNF in the hippocampal neurons [71]. Furthermore, SIT elevates beta-hydroxybutyrate to induce a BDNF-mediated fatigue mechanism, which may contribute to BDNF-induced fatigue and affect the mood of the individual performer [72,73,74].

## 5. Factors Affecting SIT Performance for BDNF Response

**Figure 3:**
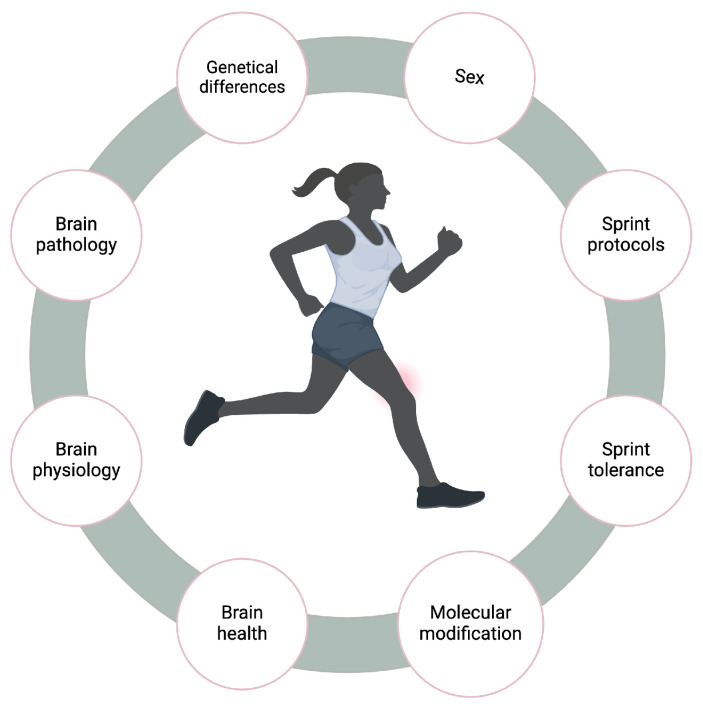
Factors affecting SIT performance for BDNF response.

Previous research has extensively examined the current state of knowledge regarding sprint training. This is crucial as it provides essential guidance for conducting high-quality, controlled sprint training to support BDNF-mediated brain health. Several factors can influence the BDNF response to sprint training, including genetic variations, sex, sprint protocols, and sprint training tolerance (Figure 3). For example, genetic differences among individuals with specific molecular modifications may affect sprint performance. This is evidenced by the increased level of AMPK activity or phosphorylation during or immediately after exercise, but it returns to the resting condition within 3 h of the exercise recovery period [75]. In contrast, protein translation signaling, like eukaryotic elongation factor 2, is suppressed during exercise but can increase over the next 24 h of recovery [76]. Similarly, PGC-1α is greatly increased for the first 4 h of recovery and returns to its baseline level within approximately 10 h [77]. Many genes are expressed in a different time course after exercise for this molecular modification [77]. Understanding this scenario may provide insight into how these molecular signals affect BDNF response to SIT. Additionally, sex differences can impact the BDNF response to SIT performance, as evidenced by women experiencing an increased BDNF response under certain aerobic exercise conditions compared to men [78]. However, specific sex-dependent signaling for BDNF activation during SIT requires further investigation. Furthermore, sprint training is pivotal for enhancing exercise tolerance, and the mechanisms by which this exercise-induced BDNF response contributes to increasing SIT limits are not fully elucidated. It is also important to consider how sprint training may negatively influence BDNF/TrkB signaling, potentially decreasing BDNF and TrkB or their downstream effects, which could contribute to brain pathology. Studies have shown that BDNF dysregulation plays a crucial role in bipolar disorder and schizophrenia [79]. For example, pro-BDNF interacts with the p75 receptor for apoptosis and neuronal shrinkage [80], while mature BDNF interacts with TrkB for neural plasticity [80]. Both pro-BDNF and m-BDNF work parallel to promote neuronal networking and neuronal remodeling, which is crucial for fostering antidepressant effects [79,80]. Understanding how SIT affects both p75 and TrkB receptors for BDNF interaction may help alleviate the symptoms of these disorders. Moreover, the beneficial effects of SIT on brain health needs a thorough exploration to mitigate potential side effects, as the effects of SIT-induced changes on brain physiology may differ from those associated with pathological alterations. For example, high intensity increases systemic blood pressure, which may be transmitted to the brain. Consequently, this could potentially elevate the risk of hyperperfusion injury in stroke [81]. These effects can manifest at various levels, from influencing the brain’s functional capacity to molecular levels, and evaluating the role of the BDNF’s response at each stage could effectively enhance our understanding of how sprint-induced BDNF response mediates brain physiology rather than inducing pathology.

## 6. Future Direction

Existing research has primarily examined how exercise can enhance the BDNF response in the brain. However, specific exercise protocols aimed at improving the BDNF response, particularly the impact of muscle contraction-induced circulating BDNF on local BDNF synthesis in the brain under specific exercise regimens, have not been developed to date. Furthermore, it remains unclear whether exercise increases the circulating BDNF to influence its response in the brain and what specific source of BDNF release can impact the local BDNF response. The mechanisms through which exercise orchestrates this scenario and the molecular targets involved under specific exercise protocols like SIT are yet to be fully elucidated. In addition, how exercise-like lifestyle changes keep maintaining the BDNF response or activating its receptors throughout life can have a potential impact on ameliorating neurodegenerative diseases like Alzheimer’s or Parkinson’s disease, which requires further understanding. Furthermore, how SIT is linked with stimulating internal networks that can impact the circulating BDNF needs to be explored. For example, internal networks like ERK are linked to brain aging and Alzheimer’s disease, and several studies have observed that various exercise types can stimulate ERK signaling in cerebral ischemia [82,83]. In addition, other signaling molecules, like ephrin-A, are linked with brain development and plasticity by inducing crosstalk with the BDNF, and this scenario can be orchestrated in ERK signaling for various gene activities [63,84]. This should also be considered under various exercise regimens. For instance, understanding how SIT-induced alteration on the molecular landscape (long term) can cause crosstalk with BDNF-mediated ephrin in the neighboring cells could drastically affect the functionality of brain tissues. Possibly, many surface receptors could be involved in this scenario, such as Fas, ErhB2, TrKB, and P75NTR [85]. Next, understanding how exercise impacts the heritability of life could favor increasing blood BDNF concentrations because blood BDNF levels are implicated with the hereditary conditions of humans, and several single nucleotide polymorphisms (SNPs) are linked with the BDNF levels in the serum. For example, wheel-running activity regulates genes like dopamine receptor 1, Nhlh2, and MC4R, which are crucial for weight regulation and improving mood [85]. In addition, focusing on SNP rs6265 is a single nucleotide substitution that disrupts BDNF secretion and transport and affects the BDNF functions. Consequently, it causes neurocognitive impairments. Studies have shown that high-intensity exercise improves locomotor learning by influencing SNP rs6265, which converts valine to methionine at codon 66 of proBDNF [86,87]. At the same time, moderate intensity possesses a protective effect in reducing depression by impacting SNP rs6265 for BDNF secretion. Therefore, targeting the SNP rs6265 and its downstream effects on BDNF through exercise regimens of varying intensities may offer promising avenues for improving mood, cognitive function, and overall mental well-being. As mentioned, this particular variant disrupts BDNF secretion and transport and affects its functions, causing neurocognitive impairments. Exploring how SIT influences SNPs as well as BDNF activity could reveal substantial evidence of SIT-induced BDNF responses on brain health. At the molecular level, there is limited evidence supporting the transport of BDNF and TrkB within neuronal cells, as well as how the BDNF’s effects are mediated by its downstream signaling. Exploring the balance of these signals at fast and slow paces, along with their corresponding mediators, in various exercise regimens is crucial. Additionally, BDNF effects in the synapses largely depend on calcium release. Understanding how calcium stores can be activated by BDNF/TrkB can help elucidate the usefulness of exercise-mediated calcium release in mediating BDNF/TrkB activation and vice versa. Lastly, advancements in the BDNF’s neurobiology can shed light on how states, both pre- and post-BDNF, can effectively yield benefits in individual synapses. It is possible that exercise could be one of these developments, effectively helping to address the challenge of understanding the fundamental questions of the BDNF’s effects in neurobiology.

## 7. Conclusions

This review discusses the potential impact of sprint training on increasing the BDNF response through various molecular signaling pathways. SIT primarily triggers AMPK and PGC-1α to promote BDNF response through sestrin to improve neuronal migration and neurogenesis in the brain. Additionally, SIT may enhance the BDNF response through PKA/AKT/CREB and MAPK/CREB, PLCγ, PI3/AKT, ERK/CREB, and p75^NTR^ pathways to improve neuronal survival. Conversely, SIT could potentially reduce the BDNF response by activating TORC2/AKT, which inhibits the PGC-1α-induced BDNF response. This could reduce the negative effect of the BDNF on bipolar disorder. Moreover, a higher BDNF response may influence the mood of SIT participants by increasing beta-hydroxybutyrate levels.

## Figures and Tables

**Table 1:** The study characteristics of SIT-induced BDNF response.

Study Population	Study Design	Sex	No of Participants	Sample Type	Age Range	Exercise Protocols	Conclusion	References
**UK**	RCT	Female	16	Serum	11.7 ± 0.3 years	Two weeks of sprint training with 3 sessions, each consisting of 10 s of maximum effort sprints. Each session lasts 6 min with 50 s of passive recovery. The final session consists of 8 min per session.	BDNF ↑	[16]
**Australia**	RCT	Male	8	Serum	53–64 years	The mixed training program involved 12 weeks of combined aerobic and resistance training. During aerobic training, participants worked at maximal effort for 30 s with an intensity of over 80%. The resistance training included 2 to 4 sets of 8 exercises for 12 weeks.	BDNF ↓	[17]
**Polish**	RCT	male	36	Serum	21.7 ± 1.3 years	30 s sprint cycling with a rest of 4.5 min.	BDNF ↑	[18]
**Lithuania**	RCT	Male	10	Serum	22.6 ± 5.2 years	Sprint interval cycling consists of 12 repeats of 5 s on a cycle ergometer.	BDNF ↓	[19]
**Canada**	RCT	Male	8	plasma	23.1 ± 3.0 years	SIT performed as four 30 s bouts of running training, interspersed with 4 min rest periods	BDNF ↑	[20]
**Qatar**	RCT	Male	21	Serum	29.8 ± 5.9 years	Sprint training (running) consisted of 5 × 5 s maximal sprints, 25 s recovery/sprints, and 3 min recovery/sets.	BDNF ↑	[21]

Study characteristics of the SIT-induced BDNF response for neuroprotection. According to the SIT program, different age groups, from 11 to 64, produced BDNF responses. RCT = randomized controlled trials. ↑= Increase of BDNF. ↓= Decrease of BDNF

**Table 2:** Proteins that promote and antagonize BDNF response in the brain.

Proteins Promote an Increase in BDNF	Signaling Pathways Promote BDNF Response	Proteins that Antagonize BDNF	Signaling Pathways Antagonize BDNF Inhibition
**Sestrin 2**	AMPK that mediates PGC-1α-induced BDNF response	EphrinA5	Ephrin-A5 traps the ERK through c-Fos, Egr1, and Arc for BDNF inhibition.
**MAPK**	MAPK induces BDNF response through CREB pathway	Sestrin	Sestrin-induced TORC2/AKT activation inhibits PGC-1α to antagonize BDNF
**CREB**	CREB increases the BDNF response through SNAP-25 phosphorylation	p75^NTR^	Decreases the Pro-BDNF response
**PDK1**	PDK1 increases BDNF via RSK1/2	AMPK	Decreases BDNF through mTOR
**PGC-1 alpha**	AMPK mediates PGC-1 alpha activation for BDNF response	NMDAR	Decreases the pro-BDNF response

AMPK—AMP-activated protein kinase; MAPK—mitogen-activated protein kinases; PGC-1α—peroxisome proliferator-activated receptor gamma coactivator 1-alpha; ERK—extracellular signal-regulated kinases; CREB—cyclic AMP response element binding protein; PDK1—phosphoinositide-dependent kinase-1; mTOR—the mammalian target of rapamycin; NMDAR—*N*-methyl-D-aspartate receptor; Egr1—early growth response protein 1; Arc—activity-regulated cytoskeleton-associated protein; and RSK1/2—ribosomal s6 kinase.

## Data Availability

Not applicable.
